# *In Silico* Scrutiny of Genes Revealing Phylogenetic Congruence with Clinical Prevalence or Tropism Properties of *Chlamydia trachomatis* Strains

**DOI:** 10.1534/g3.114.015354

**Published:** 2014-11-05

**Authors:** Rita Ferreira, Minia Antelo, Alexandra Nunes, Vítor Borges, Vera Damião, Maria José Borrego, João Paulo Gomes

**Affiliations:** * Reference Laboratory of Sexually Transmitted Bacterial Infections, Department of Infectious Diseases, National Institute of Health, Av. Padre Cruz, 1649-016 Lisbon, Portugal; † Bioinformatics Unit, Department of Infectious Diseases, National Institute of Health, Av. Padre Cruz, 1649-016 Lisbon, Portugal

**Keywords:** *Chlamydia trachomatis*, genomics, clinical prevalence, tropism, loci

## Abstract

Microbes possess a multiplicity of virulence factors that confer them the ability to specifically infect distinct biological niches. Contrary to what is known for other bacteria, for the obligate intracellular human pathogen *Chlamydia trachomatis*, the knowledge of the molecular basis underlying serovars’ tissue specificity is scarce. We examined all ~900 genes to evaluate the association between individual phylogenies and cell-appetence or ecological success of *C. trachomatis* strains. Only ~1% of the genes presented a tree topology showing the segregation of all three disease groups (ocular, urogenital, and lymphatic) into three well-supported clades. Approximately 28% of the genes, which include the majority of the genes encoding putative type III secretion system effectors and Inc proteins, present a phylogenetic tree where only lymphogranuloma venereum strains form a clade. Similarly, an exclusive phylogenetic segregation of the most prevalent genital serovars was observed for 61 proteins. Curiously, these serovars are phylogenetically cosegregated with the lymphogranuloma venereum serovars for ~20% of the genes. Some clade-specific pseudogenes were identified (novel findings include the conserved hypothetical protein CT037 and the predicted α-hemolysin CT473), suggesting their putative expendability for the infection of particular niches. Approximately 3.5% of the genes revealed a significant overrepresentation of nonsynonymous mutations, and the majority encode proteins that directly interact with the host. Overall, this *in silico* scrutiny of genes whose phylogeny is congruent with clinical prevalence or tissue specificity of *C. trachomatis* strains may constitute an important database of putative targets for future functional studies to evaluate their biological role in chlamydial infections.

The observation that there are pathogenic and nonpathogenic microbes has compelled investigators to search for traits underlying their phenotypic differences. This search for the so called “virulence factors” has greatly contributed to the understanding of pathogenicity and to the elucidation of the genetic mechanisms underlying microbes’ capability to infect different cell types or organs. The notion that microbial pathogenicity relies on the interaction between a pathogen and its host (or a specific tissue), and that a virulence factor is either a microbial product or a strategy capable of causing damage to a susceptible host, can be broadly applied (Casadevall and Pirofski 2009). In this perspective, virulence factors may involve an endless list of products and mechanisms, such as toxins, adhesins, motility structures like flagella and pili, immune evasion determinants, capsules, biofilms, secretion systems, and signal transduction mechanisms (reviewed in Casadevall and Pirofski 2009). Usually, microbes carry several of these virulence factors, which work together in the process of host invasion and microbe survival.

Among pathogenic agents, several bacteria present intracellular lifestyles (obligatory or facultative). Their host-cell targets range from epithelial cells to phagocytes, like macrophages and neutrophils (Wilson *et al.* 2002), which implies that these pathogens have been developing specialized strategies that allow them, for instance, to survive within or avoid the adverse environment of the macrophage phagosome (membrane-bound vacuole) (Garcia-Del Portillo and Finlay 1995; Pizarro-Cerdá *et al.* 1997). Whereas some bacteria (*e.g.*, *Salmonella* spp, *Coxiella burnetii*, and *Cryptococcus neoformans*) are able to reside within the lysosomal vacuole, others (*e.g.*, *Chlamydia trachomatis* and *Mycobacterium* spp) need to “remodel” it to allow their survival, whereas others (*e.g.*, *Listeria monocytogenes* and *Shigella* spp) degrade the vacuole membrane to gain access to the host-cell cytosol, where they may complete their developmental cycle (Pizarro-Cerdá *et al.* 1997; Ernst *et al.* 1999). Moreover, some pathogenic bacteria are also able to infect different cell types or organs of a given host. For example, *L. monocytogenes* can cross the intestinal epithelium, the blood–brain, and fetoplacental barriers (Cossart 2011) and may cause severe septicemia and meningoencephalitis (Allerberger and Wagner 2010), whereas *Streptococcus pneumoniae* is capable of infecting the lung, the blood, and the nasopharynx (Hava and Camilli 2002).

Another example of bacteria capable of infecting different cell types is *C. trachomatis*, an obligate intracellular human pathogen that can be classified into 15 main serovars, according to the polymorphism of the gene (*ompA*) encoding the major outer membrane protein. Serovars A-C cause ocular infections that can progress to trachoma, the leading cause of preventable blindness worldwide (Burton 2007; Wright *et al.* 2008), whereas serovars D-K cause ano-urogenital infections that can evolve to cervicitis, urethritis, epididymitis (men), or pelvic inflammatory disease (women), the latter of which can lead to significant long term sequelae such as infertility and ectopic pregnancy (Peipert 2003). Finally, serovars L1-L3 are responsible for an invasive disease, the lymphogranuloma venereum (LGV), through the infection of macrophages and dissemination to regional draining lymph nodes (Schachter 1978). Despite the huge phenotypic differences among *C. trachomatis* serovars regarding tissue tropism, virulence and ecological success, little is known about the molecular factors underlying serovars’ biological uniqueness. This is mostly due to the lack of suitable animal models that mirror the human chlamydial infection *in vivo* and because *C. trachomatis* has been genetically intractable until very recently (Kari *et al.* 2011; Wang *et al.* 2011; Mishra *et al.* 2012; Nguyen and Valdivia 2012). Probably the only unequivocal demonstration of the association of a virulence factor with tropism was provided by Caldwell *et al.* (2003), who showed that an active tryptophan operon (*trpRBA*) is mandatory for any *C. trachomatis* strain to infect the genitalia. This observation also was valid for genital strains harboring an “ocular” *ompA* gene (likely inherited by recombination), excluding the serovar status as a possible tropism determinant. Nevertheless, a revision concerning the genetics beyond tropism was recently published (Nunes *et al.* 2013).

Recent phylogenetic analysis (Harris *et al.* 2012) using the complete genome of several *C. trachomatis* strains found: *i*) the segregation of strains by their cell-appetence, suggesting a coevolution with the infected tissue; *ii*) the separation of the LGV strains before the separation of the ocular and the epithelial-genital strains; *iii*) that the most prevalent serovars (E and F), which account for ~50% of all chlamydial genital infections among the heterosexual population (Nunes *et al.* 2010), clearly segregate apart from the remainder epithelial-genital strains; and *iv)* that the ocular strains probably derived from a nonprevalent genital serovar. On the other hand, the small genome (~1 Mb) of *C. trachomatis* reveals a high degree of conservation among serovars (>98%), with nearly identical pan- and core-genomes, a high coding density, and no evidence of recent horizontal gene transfer besides allelic recombination, which suggests a likely complete genetic reduction process as a result of a long-term intracellular niche adaptation process (Horn *et al.* 2004; Read *et al.* 2013). Considering this, one may speculate that the phenotypic disparities (tissue tropism, virulence and ecological success) among strains are encoded in a small number of variable genes along the *C. trachomatis* genome. Thus, given the recent availability of dozens of *C. trachomatis* fully sequenced genomes, our main goal was to scrutinize all the ~900 genes at the phylogenetic and evolutionary level in order to better understand the relationship between strains’ genetic diversity and phenotypic disparities. In this regard, after analyzing the global trends of polymorphism, we performed a detailed analysis of each gene tree topology to assess the degree of concordance between strains’ segregation and their clinical outcome and prevalence. This approach intends to identify the genes that phylogenetically contribute for the main branches (LGV, prevalent genital, nonprevalent genital, and ocular serovars) of the species tree (Harris *et al.* 2012).

## Materials and Methods

### Alignments generation

For the polymorphism and evolutionary analyses, different alignment strategies were conducted. First, the whole-genome sequences of the 53 studied *C. trachomatis* strains were retrieved from the GenBank (Supporting Information, Table S1) and aligned using progressiveMauve from Mauve software, version 2.3.1 (Darling *et al.* 2010). Orthologous genes were identified by Mauve and individual alignments of each one of the 896 genes (considering the total number of annotated genes on the available D/UW-3/CX sequence) were extracted from the whole-genome alignment. These alignments were subsequently uploaded into the Molecular Evolutionary Genetics Analysis software, version 5 (MEGA 5; http://www.megasoftware.net) (Tamura *et al.* 2011) and visually inspected for further correction (whenever needed) prior to evolutionary and genetic diversity analyses. A core-alignment was also extracted by keeping regions where the 53 genomes aligned over at least 500 bp (corresponding of ~97% of the *C. trachomatis* chromosome), and aligned segments were concatenated into a single-core genome alignment to be further used in the construction of the species phylogenetic tree. This alignment was then exported and directly uploaded into MEGA 5 for whole-genome analyses purposes.

### Exclusion criteria

Among all the 53 strains, variability in start codon predictions of homologous genes was removed by trimming each start site prediction to the innermost common start codon. This was not applied when an upstream codon was annotated as a consequence of a mutation in the codon correspondent to the translation initiation codon of the other sequences. We also observed that, for some other genes, there were strains that had more than one coding sequence annotated at the same region. These cases were treated as pseudogenes and the respective strains were removed from the analysis. There were also genes for which a single frameshift yielded a biased polymorphism, and for this reason they were not considered has truly polymorphic. Nevertheless, some of them (CT120, CT160, CT162, CT172, CT172.1, CT358, CT480.1, CT793, and CT852) constitute interesting cases as the frameshift occurred solely for the strains of the same disease group. Moreover, for 22 chromosomal genes, it was not possible to obtain an accurate alignment (Table S2) mainly because of accentuated gene size differences, hampering the analyses.

### Polymorphism and evolutionary analyses

Each alignment (core-genome and individual genes) was analyzed according to previously described methods (Nunes *et al.* 2008; Almeida *et al.* 2012). Concerning the individual alignments of all homologous genes, we first removed from each analysis the strains’ sequences that were considered as putative pseudogenes or had annotation issues (see the section *Exclusion criteria*). By using the algorithms available in MEGA 5, we determined the overall mean distances (number of differences and *p*-distance) and matrices of pairwise comparisons at both nucleotide and amino acid level, along with the respective standard error estimates (bootstrap = 1000). Then, for each gene, the number of synonymous substitutions *per* synonymous site (dS) as well as the number of nonsynonymous substitutions *per* nonsynonymous site (dN) were determined by using the Kumar model (Nei and Kumar 2000) and the standard error estimates were obtained by a bootstrap procedure of 1000 replicates. dN/dS ratios were determined and the Z-test of positive selection was applied for the genes revealing dN/dS > 1. The probability of rejecting the null hypothesis of strict-neutrality (dN = dS) in favor of the alternative hypothesis of positive selection (dN > dS) was considered significant when *P* < 0.05 (bootstrap = 1000) (Nei and Kumar 2000). We also assessed the existence of correlation between *p*-distance and dN, dS, or dN/dS by using the Pearson’s Product Moment Correlation coefficient (R), which measures the strength and direction of a linear relationship between two variables (Rodgers and Nicewander 1988).

Phylogenetic trees for both the whole-genome and individual genes sequences were inferred by using the Neighbor-Joining method (bootstrap = 1000) (Felsenstein 1985; Saitou and Nei 1987). For the nucleotide sequences, the evolutionary distances were computed using the Kimura 2-parameter method (K2P) (Kimura 1980), whereas for the amino acid sequences (for individual genes solely), the evolutionary distances were computed based on the number of differences (Nei and Kumar 2000). A gene was considered to segregate a specific group of strains (ocular, genital and LGV serovars) by taking into account both the tree topology and the number of differences between sequences of different taxa. Additionally, phylogenies were also inspected for the segregation of the strains from the most prevalent genital serovars.

### Characterization of the mosaic structure of the strains from the most prevalent serovars

We started by comparing the genome sequences of both D(s)/2923 and D/SotonD1 with that of the F/SW5 strain (because this strain was found to be the most closely related to both – see Results section) using the *DNA polymorphism* tool of the DnaSP software, version 5 (Librado and Rozas 2009), with a window size and step size of 1000 each. Chromosomal regions with high SNP density, which may indicate the occurrence of recombination events, were further analyzed by SimPlot/BootScan (http://sray.med.som.jhmi.edu/SCRoftware/simplot/) (Salminen *et al.* 1995;Lole *et al.* 1999) for a precise determination of potential mosaic structures. These analyses were performed as previously described (Gomes *et al.* 2007), using a sliding window size of 200 bp moved across the alignment in a step size of 30 bp for estimating pairwise genetic distances with Neighbor-Joining method (Kimura 2-parameter method; Bootstrap = 500; gaps strip off; ts/tv of 2.0). For BootScan analyses, the likelihood that the observed distribution of informative sites (Robertson *et al.* 1995) favoring specific phylogenetic groupings might occur randomly was assessed using the maximum χ^2^ test. A *P*-value for any specified breakpoint was determined by the Fisher’s exact test (two-tailed). A Bonferroni multiple correction testing was applied to evaluate the significance of the *P*-values at 95% confidence.

## Results

### Polymorphism and molecular evolution analysis

Overall, we were able to analyze ~97.5% (874/896) of all the *C. trachomatis* chromosomal genes. The 22 genes excluded from the analysis (see the section *Exclusion criteria*) comprise five housekeeping genes, the cytotoxin locus, genes encoding 13 hypothetical proteins, two of the phospholipase D endonuclease superfamily gene members (PLDs), and CT081 (Table S2).

Besides well-known polymorphic genes (CT870/*pmpF*, CT872/*pmpH*, CT681/*ompA*, CT049-CT051), the polymorphism analyses highlighted CT619 (Table 1 and Table S2) [coding for a putative type III secretion system (T3SS) secreted protein with unknown function] that, to our knowledge, had never been considered before as polymorphic.

**Table 1:** Top five ranking of the most polymorphic *C. trachomatis* chromosomal genes

Rank	Nucleotide		Amino Acid	
	No. Differences	*p*-distance	No. Differences	*p*-distance
1	CT870/*pmpF* (217.3)	CT681/*ompA* (0.121)	CT870/*pmpF* (72.4)	CT681/*ompA* (0.107)
2	CT681/*ompA* (143.7)	CT051 (0.07)	CT619 (48.4)	CT051 (0.093)
3	CT619 (124.2)	CT870/*pmpF* (0.069)	CT051 (46.6)	CT049 (0.08)
4	CT872/*pmpH* (109)	CT049 (0.048)	CT681/*ompA* (42.1)	CT870/*pmpF* (0.071)
5	CT050 (104.9)	CT619 (0.047)	CT049 (38.9)	CT050 (0.058)

The numbers in parenthesis refer to the respective number of differences and *p*-distance value.

To understand the underlying evolutionary pressures that drove amino acid changes of all 874 analyzed chromosomal proteins, we evaluated their molecular evolution by determining the dN/dS values of the respective genes. We verified that 150 genes (~17%) revealed a dN/dS > 1, but only 31 (3.5%) showed a significant Z-test of positive selection (Table S2) and were thus considered as putative targets of positive selection. Twenty-three of the latter encode 11 Inclusion Membrane Proteins (Incs), 10 T3SS effectors, and two putative membrane proteins, which are proteins expectedly involved in interactions with the host. We also found three hypothetical proteins encoding genes, one PLD encoding gene, and four housekeeping genes that are likely under positive selection. We have no reasonable explanation for the latter finding, as housekeeping genes are usually highly conserved and expected to be under purifying selection.

**Figure 1:**
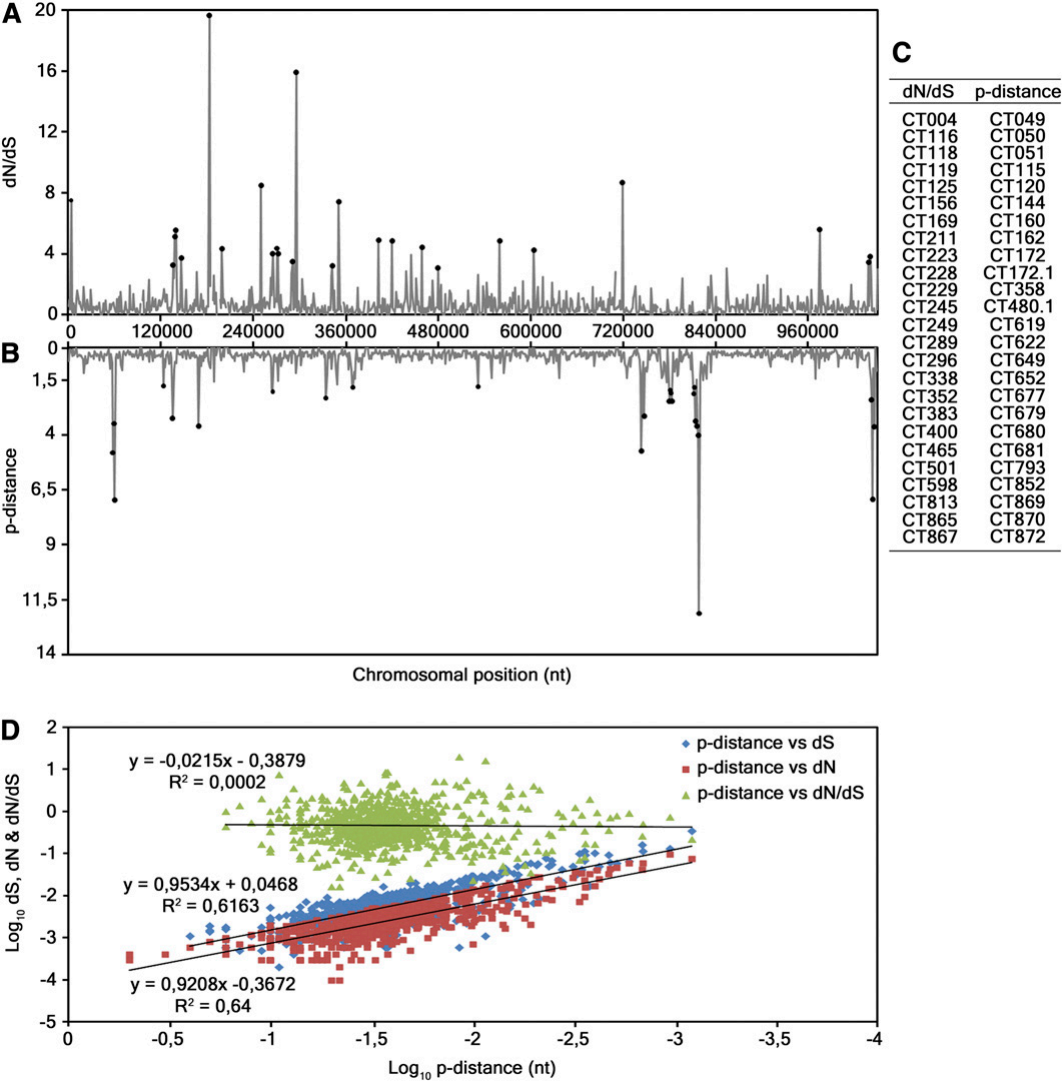
Evaluation of the association between polymorphism and dN, dS and dN/dS. (A and B) Distribution of dN/dS and *p*-distance values, respectively, obtained from the analyses of all the 874 genes from the 53 strains. The horizontal axis represents the *C. trachomatis* chromosomal positions where genes are placed in their chromosomal order, from the CT001 to the CT875 (genes names and positions according to D/UW-3/CX strain annotation). (C) 25 genes (ordered by their relative chromosomal position) that display the greater values for both analyses, which are representative of the lack of correlation between dN/dS and polymorphism. (D) Scatter plots of *p*-distance *vs.* dN, dS, and dN/dS, on a log-scale for clarity. The Pearson’s product moment correlation coefficient for *p*-distance *vs.* dN, dS, and dN/dS are R = 0.92, R = 0.9, and R = 0.02, respectively.

Furthermore, we evaluated the correlation between nucleotide polymorphism and evolutionary parameters, such as dN, dS, and dN/dS, for all 874 chromosomal genes. From the inspection of the genomic distribution of *p*-distance and dN/dS (Figure 1, A and B) and by determining the Pearson’s product moment correlation coefficient, we observed no correlation between them (R = 0.02), besides minor coincident peaks. Figure 1C highlights the 25 top ranked loci for both parameters. On the other hand, a strong positive linear correlation between *p*-distance and both dN (R = 0.92) and dS (R = 0.9) was found (Fig. 1D).

### Species polymorphism *vs.* number of taxa

**Figure 2:**
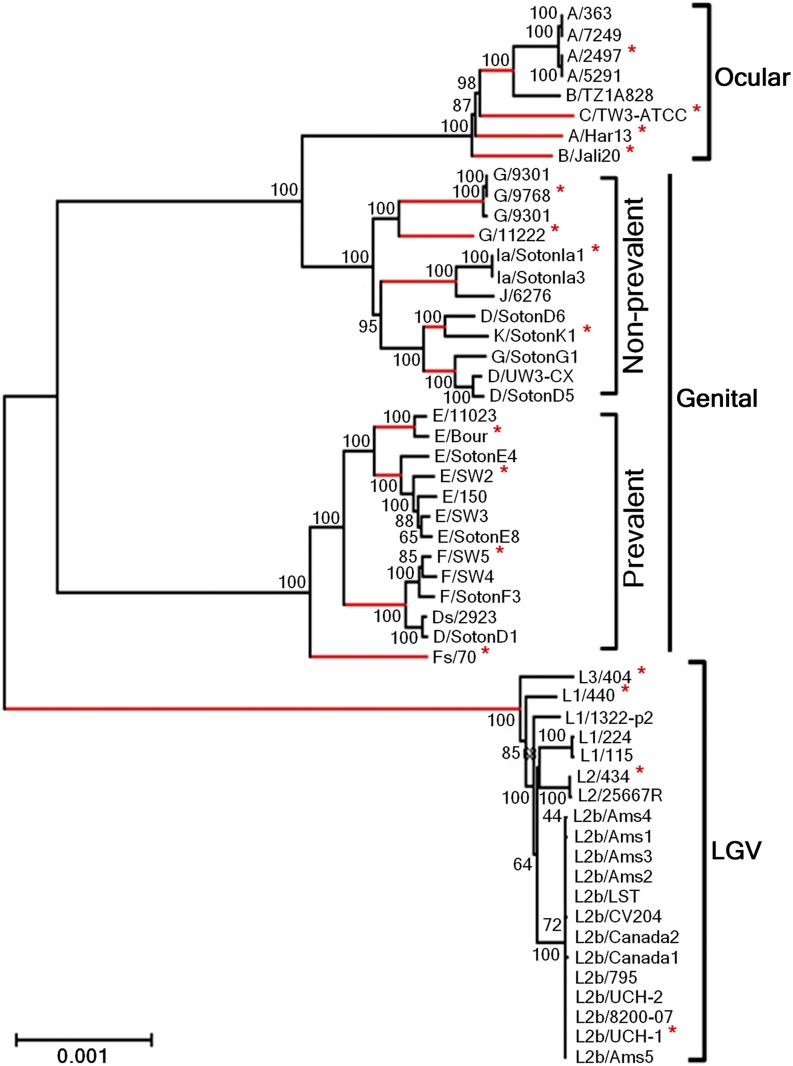
Phylogenetic reconstruction of *C. trachomatis* species. The tree was constructed using the whole genome of 53 strains encompassing the majority of the CT681/*ompA* serovars. The asterisks indicate the 17 strains representative of the major tree branches (in red) that were used to evaluate the relation between species polymorphism and the number of taxa (see the section *Results* for details).

**Figure 3:**
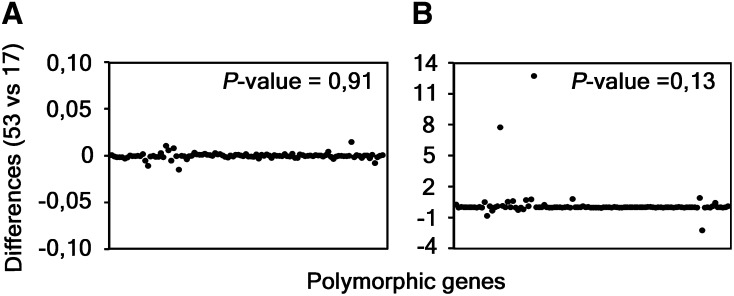
Differences obtained during the analyses using 53 and 17 strains. The graphs show the differences obtained between the results of the *p*-distance (A) and the dN/dS (B) analyses of all the 53 and the set of 17 strains (representative of the majority of the tree branches). Each black dot represents one of the 100 polymorphic genes selected for these comparisons. *P*-values were calculated through the paired two-tailed *t*-test.

Considering that the genetic diversity among same-serovar or same-disease group strains was recently pointed out to be higher than expected (Harris *et al.* 2012), we wonder whether both the polymorphism and selective pressure results are impacted by the number of sequences used. Thus, besides using all 53 strains, we also selected a group of 17 strains representative of the major branches of the phylogenetic tree constructed with the whole-genome sequences (Figure 2). Both groups of strains (17 *vs.* 53) encompass the same set of 13 *C. trachomatis* serovars. We then used the 100 most polymorphic genes (as they provide the vast majority of informative sites) and compared the distribution of polymorphism and dN/dS obtained from the analysis of the two groups (Figure 3). The *P*-values (paired two-tailed *t*-test) calculated for the *p*-distance and the dN/dS results were 0.91 and 0.13, respectively, which indicates that these parameters do not depend on the number of same-serovar sequences that are used. Although the validity of the traditional CT681/*ompA* typing has been strongly questioned (as its tree does not segregate strains by tissue tropism properties and disease outcomes) (Harris *et al.* 2012), it is worth noting that a small group of strains encompassing the majority of the *C. trachomatis* serovars represent the main genetic variability of this bacterium.

### Gene-based phylogenetic analysis

**Figure 4:**
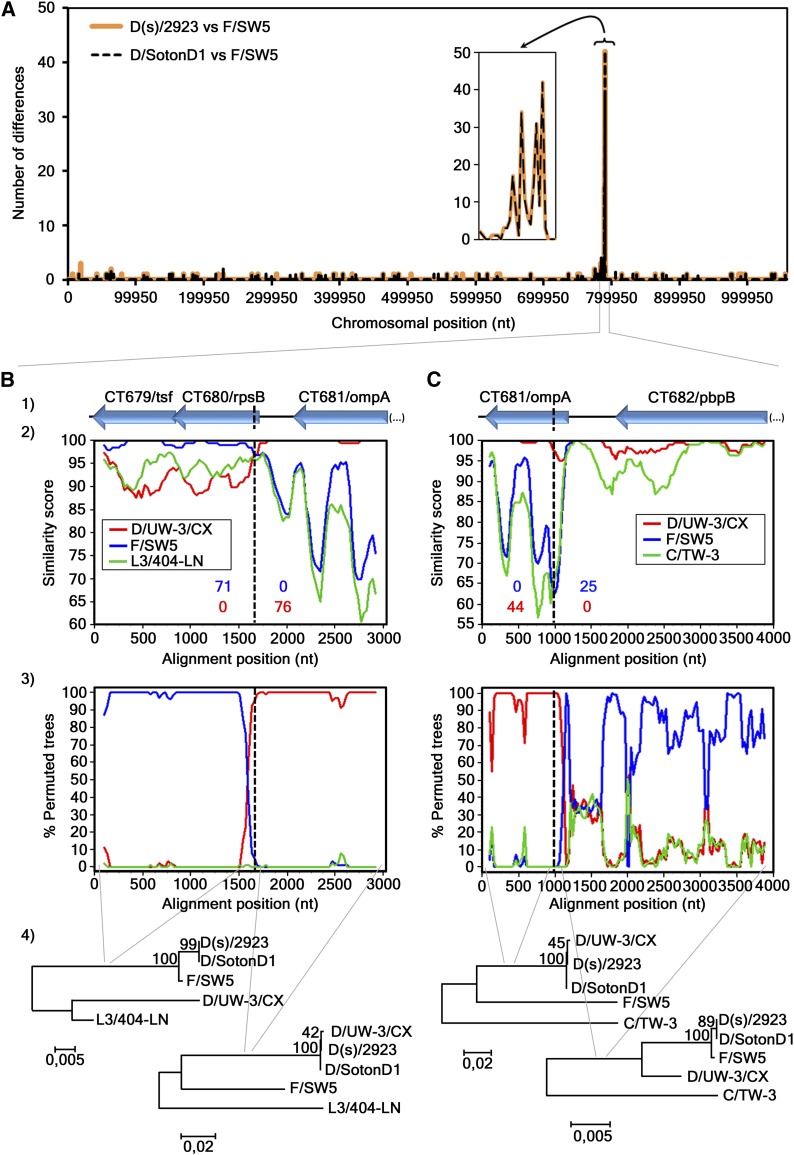
Recombination analyses of the D(s)/2923 and D/SontonD1 strains. (A) Number of nucleotide differences (vertical axis) that exist between the genomic sequence of D(s)/2923 or D/SotonD1 and F/SW5. This polymorphism assessment was performed by using the DnaSP software, v5, with a window size and a step size of 1000 base pairs each. The smaller graph represents an enlarged view of the detected highly polymorphic region. (B) (first crossover) and (C) (second crossover) show the genes in each analyzed region (1) and also the results of the SimPlot (2), the BootScan (3), and the phylogenetic (4) analyses. Recombination breakpoints were individually analyzed because they were better mapped when a different outgroup strain was used for each one, *i.e.*, the L3/404-LN for the first (B) and the C/TW-3 for the second (C) breakpoint. SimPlot graphs (2) show the level of similarity between the recombinant sequences and the respective parental strains (the number of informative sites supporting this relatedness are colored according to the graph legend box), whereas the BootScan graphs (3) show the phylogenetic relatedness (% of permuted trees) between those same sequences. Both analyses were obtained with a sliding window size of 200 bp and a step size of 30 bp. The sequence of the recombinant D strains was used as query. The vertical dashed black lines indicate the location of the estimated crossovers, shown in detail in Figure S1. Seventy-one informative sites support the similarity between the recombinant strain and F/SW5, whereas 76 support its similarity with D/UW-3/CX (*P* = 9.28 × 10^−44^). Forty-four informative sites support the similarity between the recombinant strain and D/UW-3/CX, whereas 25 support its similarity with F/SW5 (*P* = 6.65 × 10^−19^). In these defined regions there are no informative sites supporting the alternative hypotheses. The phylogenetic trees (4) were constructed with the nucleotide sequences adjacent to each estimated breakpoint region (NJ method; Kimura 2-parameter method; bootstrap = 1000) and support the recombination event.

To evaluate the concordance between strains’ segregation and their clinical outcome and prevalence, we first performed a detailed analysis of the recombination phenomena involving the two D strains (D(s)/2923 and D/SotonD1) that phylogenetically cluster with the most prevalent serovars (E and F) and apart from the other D strains (Jeffrey *et al.* 2010; Harris *et al.* 2012), in order to define their true genomic backbone. We verified that those D strains differ from the same serovar prototype strain (D/UW-3/CX) by ~5500 nucleotides, but differ from a serovar F strain (F/SW5) by only ~300 nucleotides, with ~50% of these mutations concentrated at the CT681/*ompA* region (Figure 4A). SimPlot and BootScan analyses identified the exact location of the two breakpoints underlying the recombination event (identical for both strains) (Figure S1). One breakpoint is located at the beginning of CT680/*rpsB* (*P* = 9.28 × 10^−44^) (Figure 4B), whereas the other is located at the beginning of CT681/*ompA* (*P* = 6.65 × 10^−19^) (Figure 4C). These results clearly indicate that both recombinant D strains have a genome backbone of a serovar F strain, whereas solely the region spanning between the two recombination breakpoints was inherited from a serovar D strain. Therefore, from now on these two D serovar strains will be included into the cluster of the most clinically prevalent serovars.

**Figure 5:**
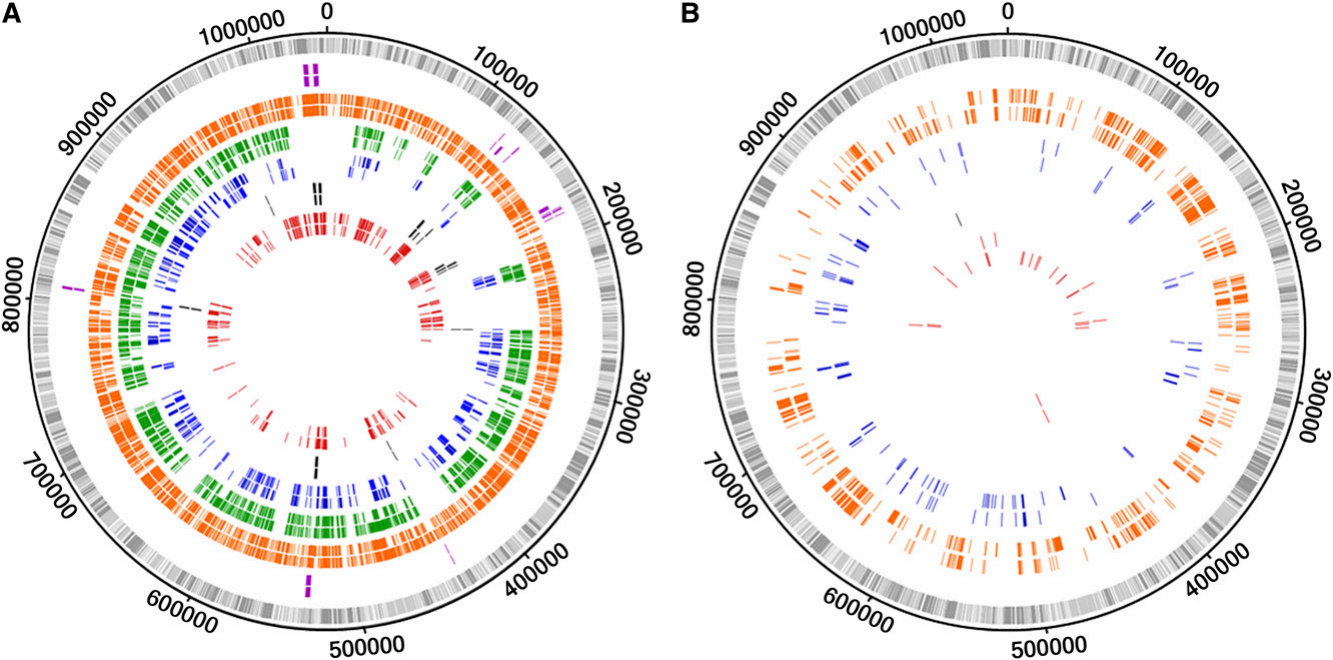
Genes that segregate strains according to their biological characteristics. The outer circle in both panels represents the genome of *C. trachomatis* D/UW-3/CX strain, where each bar represents a gene at its respective genomic position (light gray bars, forward strand; dark gray bars, reverse strand). (A) The tracks’ color scheme represent genes whose phylogeny segregates at least a group of strains according to their biological characteristics, *i.e.*, each color illustrates a particular segregation (that may not be exclusive): full-tropism (purple), LGV strains (orange), strains from prevalent genital serovars (green), cosegregation of LGV and prevalent genital serovar strains (blue), genital strains (prevalent and nonprevalent serovars) (black), and ocular strains (red). (B) The tracks’ color scheme was maintained for the different groups of strains and represent genes that exclusively segregate a unique group of strains. For both panels, the outer and inner tracks of each color correspond to nucleotide and amino acid results, respectively.

To identify loci that phylogenetically contribute for the main branches of the species tree (Harris *et al.* 2012), we performed a detailed analysis of each gene phylogenetic tree. For clarification purposes, a gene/protein was considered to segregate a group of strains sharing a specific phenotype (ocular, prevalent genital, non-prevalent genital and LGV serovars) when the genetic differences among them are lower than the differences to any other strain. Overall, we found that 136, 14, 431, and 695 genes phylogenetically segregate the ocular, genital, prevalent genital and LGV groups, respectively (Figure 5A, Table 2, and Table S2). The low number of genes segregating the group of genital serovars reflects the high heterogeneity within this group as a direct consequence of the recombination background affecting mostly these strains (Harris *et al.* 2012) and the existence of distinct polymorphism signatures. An example of the latter stands for the F(s)/70 strain, which was isolated from the cervix and frequently showed a rather unusual polymorphism pattern that did not resemble any of the other 52 strains. Therefore, only 11 (1.3%) of nucleotide trees and 12 (1.4%) of protein trees were found to segregate strains by full-tropism (Figure 5A and Table 2), where ocular, LGV and all genital (prevalent and nonprevalent) serovar strains are segregated into three main clusters. *In silico* studies have already implicated some of these genes in the different cell-appetence of the strains, namely CT456/*tarp*, CT870/*pmpF*, CT872/*pmpH*, CT115/*incD*, CT116/*incE*, two PLD (CT156 and CT157), and one MACPF domain family protein (CT153) (Gomes *et al.* 2006; Thomson *et al.* 2008; Borges *et al.* 2012; Lutter *et al.* 2012). The remainders include three housekeeping genes (CT106/*yceC*, CT110/*groEL1*, and CT703/*engA*), and genes encoding one T3SS effector (CT161) (da Cunha *et al.* 2014) and one putative inclusion membrane protein (Inc) (CT383) (Dehoux *et al.* 2011) (Table S2).

**Table 2:** Number of genes/proteins that segregate *C. trachomatis* strains according to distinct phenotypes

	Segregation by Phenotype*^a^*						Exclusive Segregation by Phenotype*^b^*			
	Full-tropism***^c^***	Ocular	Genital*^d^*	Prevalent Genital	Prevalent Genital + LGV*^e^*	LGV	Ocular	Genital*^d^*	Prevalent Genital	LGV
Nucleotide	11 (1.3%)	136 (15.6%)	14 (1.6%)	431 (49.3%)	173 (19.8%)	695 (79.5%)	7 (0.8%)	0 (0%)	47 (5.4%)	245 (28%)
Amino acid	12 (1.4%)	105 (12%)	15 (1.7%)	302 (34.6%)	146 (16.7%)	531 (60.8%)	21 (2.4%)	1 (0.1%)	61 (7%)	240 (27.5%)

The numbers in parenthesis refer to the proportion of genes/proteins, found in each category, relative to the 874 analyzed genes/proteins. LGV, lymphogranuloma venereum.

a Genes/proteins for which the phylogenetic tree differentiates at least one group of strains in a nonexclusive manner.

b Genes/proteins for which the phylogenetic tree differentiates only one particular group of strains whereas the remainder are mixed.

c Genes/proteins for which the phylogenetic tree shows three clades (ocular, genital, and LGV serovars).

d Refers to all genital strains (prevalent plus non-prevalent serovars).

e Genes/proteins for which the phylogenetic tree clusters the strains from prevalent genital and LGV serovars in the same clade.

We also detected events of exclusive phylogenetic segregation, *i.e.*, the clustering of a particular group of strains sharing the same phenotype, whereas the remainder strains (regardless of their phenotype) are mixed together. For instance, the group of strains from the most prevalent genital serovars (E, F, and recombinant D strains) are exclusively segregated by 61 proteins, which may contain molecular features that contribute for their higher ecological success. We also observed that the most prevalent and the LGV serovars share hundreds of mutations, particularly in 173 genes (Table 2) revealing a major tree branch where these two groups co-segregate apart from the remaining strains. Concerning the LGV group, ∼28% of all chromosomal genes exclusively segregate these strains (Figure 5B), conferring this group a unique genetic make-up within the species diversity.

Also, based on either the presence of nonsense mutations or the considerable differences in gene size, we scrutinized the genome for the existence of genes that are putative pseudogenes exclusively for a specific disease group (Table 3). This set includes: *i*) CT058 [a putative Inc (Almeida *et al.* 2012)], CT105 [a T3SS effector possibly involved in the cell-appetence of the genital strains (Borges *et al.* 2012; da Cunha *et al.* 2014)], *trpRBA* operon (Caldwell *et al.* 2003), and CT374/*aaxC* (Giles *et al.* 2009), which are pseudogenes for most ocular strains; *ii*) CT101 [Inc (Almeida *et al.* 2012)] is a pseudogene for the majority of the genital strains; *iii*) CT473 (predicted α-hemolysin) is a pseudogene for the prevalent genital serovar strains; *iv*) CT373/*aaxB* (Giles *et al.* 2009) and CT300 [putative Inc (Dehoux *et al.* 2011)] are pseudogenes for LGV strains (Almeida *et al.* 2012) (for CT300, this occurs only if one considers the same start codon as that annotated for ocular and genital strains); and *v*) CT037 (conserved hypothetical protein) is a pseudogene for both prevalent genital and LGV serovars strains. This scenario suggests that these genes may be expendable for the *C. trachomatis* infection of specific biological niches.

**Table 3:** *C. trachomatis* known and putative pseudogenes for a particular disease group and genes that present differences in gene length among strains from different disease groups

Gene	Functional Category	Strains Group				Observations	Reference
		Ocular	Nonprevalent Genital	Prevalent Genital	LGV		
CT037	HP	=	R	Ψ	Ψ	A/2497, A/363, A/5291, and A/7249 are smaller than the nonprevalent genital serovars.	This study
CT052	Coproporphyrinogen III oxidase	>	>	=	R		This study
CT058	Putative inclusion membrane protein	Ψ	=	=	R	A/Har13 and C/TW-3 are not Ψs.	Almeida *et al.* 2012
CT101	Inclusion membrane protein	=	Ψ	Ψ	R	E/Bour, E/11023, and D/UW3 are not Ψs.	Almeida *et al.* 2012; This study
CT105	T3S effector	Ψ	=	=	R		Borges *et al.* 2012
CT106	Predicted pseudouridine synthetase family	=	>	>	R		This study
CT160	HP	>	>	>	R	B/Jali20 is a Ψ, and F(s)/70 is smaller.	This study
CT161	HP	=	<	<	R	B/Jali20 and E/SotonE8 are Ψs.	This study
CT162	HP	<	<	<	R	E/SotonE8, F(s)/70, J/6276, Ia/SotonIa1, and Ia/SotonIa3 are Ψs.	This study
CT171	Tryptophan synthase (alpha chain)	Ψ	=	=	R	B/TZ is not a Ψ.	Caldwell *et al.* 2003
CT172	HP	<	<<	<<	R		This study
CT234	Membrane transport protein from the major facilitator superfamily	=	=	<	R		This study
CT300	Putative inclusion membrane protein	R	=	=	Ψ		Almeida *et al.* 2012
CT358	HP	>	>	>	R	B/Jali20 is smaller.	Almeida *et al.* 2012; Thomson *et al.* 2008
CT373	HP	R	=	=	Ψ		Giles *et al.* 2009; This study
CT374	Arginine/ornithine antiporter	Ψ	=	=	R		Giles *et al.* 2009; This study
CT392	HP	>	>	>	R		This study
CT441	Tail-specific protease	<	<	=	R	Ia/SotonIa1, Ia/SotonIa3, and J/6276 have the size of the LGV and prevalent genital serovars sequences.	This study
CT470	HP	=	=	>	R		This study
CT473	HP	=	=	Ψ	R		This study
CT480.1	HP	>	>	>	R	G/9301, G/9768, G/11074, J/6276, Ia/SotonIa1, and Ia/SotonIa3 are smaller than the remainder strains’ sequences.	This study
CT522	S3 ribosomal protein	=	=	<	R		This study
CT605	HP	>	>	=	R		This study
CT793	HP	>	>	>	R	G/9301, G/11074, and G/9768 are Ψs.	This study
CT807	Glycerol-3-P acyltransferase	<	<	=	R		This study
CT809	HP	<	<	=	R		This study
CT833	Initiation factor 3	<	<	<	R		This study
CT852	YhgN family	<	<	<	R		This study
CT868	Membrane thiol protease (predicted)	>	>	>	R		This study

The differences in sequence length shown only refer to differences in termination between strains. Genes with discordant 5′ annotation, for which the correct start codon lacks confirmation, were not included. The differences in length do not contemplate indel events. LGV, lymphogranuloma venereum. Ψ, sequences considered as pseudogenes; R, the sequence whose size was used for reference purposes. LGV sequences were used by default except for LGV pseudogenes; =, gene of the same size as the reference; >, gene larger than the reference; <, gene smaller than the reference; <<, gene with the smallest size. Three sequence sizes were observed for CT172, depending on the disease group.

## Discussion

Phylogenetic studies in *C. trachomatis* have been extensively performed on dozens of genes. Given the recent availability of more than 50 genomes, we sought to perform comparative genomics to examine all the ~900 *C. trachomatis* genes. We aimed to evaluate the degree of concordance between strains’ segregation and their clinical outcome and prevalence. In fact, the molecular basis underlying tissue specificity in *C. trachomatis* remains to be elucidated, although it is believed that it may rely on SNPs or small *indel* events in specific genes (Nunes *et al.* 2013), given the tremendous genome similarity (> 98%) among sequenced strains. It is known that there are biases associated with phylogenetic-based inferences (“phylogenetic dependence”), such as the weight of neutral mutations in the tree topology. Nevertheless, there are well-built examples in the literature where tree topology of *C. trachomatis* genes seems to be associated with niche specificity. This is the case of Tarp (Translocated actin-recruiting protein), for which distinct functional domains that are variable in number across serovars from different disease groups likely lead to differences in the host-cell actin-driven uptake of *Chlamydia* and to differential activation of diverse signaling pathways (like the Rac/WAVE2/Arp2/3 cascade and the humoral and cellular immune signaling pathways) (Clifton *et al.* 2004; Mehlitz *et al.* 2010; Carabeo 2011). Another relevant example is provided by most Incs, which may be associated with infection of mononuclear phagocytes due to the existence of specific mutational patterns leading to the phylogenetic segregation of LGV strains and to the higher expression of some Incs in these strains (Almeida *et al.* 2012; Lutter *et al.* 2012). In this regard, although our phylogenetic approach certainly carries associated biases, the identification of genes that phylogenetically contribute for the main branches (LGV, prevalent genital, non-prevalent genital and ocular serovars) of the species tree may be highly relevant for future functional studies.

Overall, only ~1.4% (12/874) of the proteins was found to present a plain segregation of strains according to their tissue tropism (ocular conjunctiva, genital epithelium, and lymph nodes). This low number is probably due to the existence of intra- and intergenic recombination events that take place during mixed infections [believed to occur at a frequency of approximately 1% (Clarke 2011)], essentially involving the genital strains (Harris *et al.* 2012). Although *C. trachomatis* is known to have a low population-level recombination rate based on the frequency and relative weight of recombination and mutation events (Joseph *et al.* 2011; Ferreira *et al.* 2012; Joseph *et al.* 2012), recombination has been detected, even among different disease-causing strains, and hotspots have been identified (Gomes *et al.* 2007; Harris *et al.* 2012). The biological role of some of these proteins has already been assessed (Nelson *et al.* 2006; Taylor *et al.* 2010; Derré *et al.* 2011), but with exception of the above cited Tarp, only a single serovar was tested, hampering any implication in tropism. On the other hand, it is possible that each of the corresponding genes is simply evolving more quickly than the genome average (quite likely due to host pressures).

A radically different scenario is found for the lymph nodes niche, as the majority of the genes (~80%) segregate the LGV strains and 28% (245/874) segregate them in an exclusive manner (Figure 5 and Table 2). This corroborates the early divergence of these strains (Stephens *et al.* 2009) and/or their fastest evolutionary nature. The latter hypothesis may rely on the fact that the LGV strains must be capable of establishing a wider set of molecular interactions and be subject to additional selective pressures, given their ability of infecting two distinct cell-types (epithelial and mononuclear phagocytes). It is worth noting that the majority of the genes encoding T3SS effectors and Incs (known and putative) segregate the LGV strains. One interesting example is CT144 that codes for a putative substrate of the T3SS (da Cunha *et al.* 2014) and is likely involved in the “men who have sex with men” epidemiological sexual network (Christerson *et al.* 2012), for which most of LGV-specific polymorphisms are concentrated in ~150 bp on the first half of the gene (Nunes *et al.* 2008), highlighting this specific region as the one hypothetically involved in the interaction with the host cell. Another example comes from the well-studied T3SS effector Tarp for which the enhanced phosphorylation found in LGV strains was shown to additionally promote high affinity interactions with proteins associated with the immune signaling pathways (Mehlitz *et al.* 2010), likely explaining the capacity of these strains to cross the mucosa epithelium and to infect mononuclear phagocytes.

We also observed that half of the *C. trachomatis* genes segregate strains of the most prevalent genital serovars, where 61 encode proteins displaying a mutational pattern that is exclusive of these strains. The majority of these genes (33/61) encode proteins that mediate basic cellular functions, like some redox reactions (CT078/*folD*, CT278/*nqrB*, CT539/*trxA*, and CT745/*hemG*), structural ribosomal proteins (CT125/*rplM*, CT506/*rplQ*, CT511, CT523/*rplV*, CT525/*rplB*, and CT787/*rpsN*) and proteins intervenient in the translation process (CT193/*tgt*, CT437/*fusA*, and CT851/*map*). However, given the high representation of these functional categories in *C. trachomatis* genome, we can hardly assume that specific metabolic functions underlie the higher clinical prevalence of strains from serovars E and F. Nevertheless, it seems clear that these serovars share a singular genomic makeup. In fact, two recombinant strains classified as serovar D that cluster in the same branch as E and F are actually F-like strains, and so, the branch of the most ecological succeeded serovars involve exclusively taxa with E or F backbone.

Curiously, we also found that 173 genes (19.8%) cosegregated the strains from the most prevalent genital serovars and the LGV strains. Some relevant examples refer to CT651, a possible virulence factor since it is under the regulation of *C. trachomatis* plasmid (Song *et al.* 2013), and CT338 and CT619, two T3SS substrates (Muschiol *et al.* 2011; da Cunha *et al.* 2014). Possible explanations for the existence of hundreds of shared polymorphisms between these two groups include: *i*) incomplete lineage sorting, where several unresolved polymorphisms would have been accumulated in the common ancestral before the clades’ separation of the current species tree (Galtier and Daubin 2008); *ii*) recombination, although it cannot fully explain this scenario as the genetic exchange between these two groups has been recently demonstrated to be restricted to limited genomic regions (Harris *et al.* 2012); and *iii*) short coevolutionary process between E/F and LGV strains before the separation of the latter. One may speculate that some of the shared polymorphisms could endow “invasive” properties to the most prevalent serovars strains. If that would be the case, it would mirror for instance the infection scenario of *L. monocytogenes*, which is capable of surviving within macrophages and also replicating in a variety of nonphagocytic cells (Pizarro-Cerdá *et al.* 2012). Therefore, one could hypothetically identify E or F strains during recent LGV outbreaks in Europe and USA. However, full-genome sequencing was not performed for all strains identified in those outbreaks and, to our knowledge, no E and F strains were identified so far. Thus, no immediate assumption can be made concerning specific phenotypes conferred by the related mutational pattern in those 173 genes between E/F and invasive strains.

We also identified several putative pseudogenes occurring in different strains (Table 3). The most interesting cases were the genes that were truncated only for strains of the same disease group, as it may be an indication of their expendability for the infection of a specific niche. We highlight the CT473, a lipid droplet-associated protein (Lda3) found to be translocated into the host cell cytoplasm and capture lipid droplets (Kumar *et al.* 2006), which is likely being lost on the course of the evolutionary process of the strains from prevalent genital serovars, and the CT037 (conserved hypothetical protein), which is a pseudogene in both the prevalent genital and LGV serovar strains. Although we have no clues about the importance of maintaining a functional protein in the clades where these genes are not pseudogenes, it was already demonstrated for example that a functional *trpRBA* operon is mandatory for any strain to infect the genitalia (Caldwell *et al.* 2003). Also, we have previously shown that the positively selected gene CT105 (a pseudogene for ocular strains) has a significant overrepresentation of nonsynonymous mutations when comparing sequences between urogenital and LGV strains (Borges *et al.* 2012), indicating that it has been evolutionarily diverging toward niche-specific adaptation. The identification of niche-specific pseudogenes may be indicative that further genome reduction may still be ongoing in *C. trachomatis*, leading to the future disappearance of those sequences from the chromosome. We also identified genes with differences in sequence length according to strains phenotype. For instance, both CT833 (translation initiation factor) and CT852 (integral membrane component) have longer sequences for all LGV strains, making them interesting targets for future evaluation, as the gene length may have a differential impact on the protein functionality. Additional analyses are now being performed at our lab in order to better characterize this complete set of genes (Table 3).

The analysis of polymorphism and dN/dS revealed no correlation between the two parameters, indicating that positive selection is highly targeted on specific genes or gene regions, or acts on strains with specific cell-appetence (Borges *et al.* 2012). Although our analysis was focused on whole genes (leading to an underestimation of positive selection), it is notable that the genes with significant dN/dS > 1 were mainly *incs* and T3SS effectors encoding genes, whereas the most polymorphic ones code essentially for membrane and hypothetical proteins. This seems to corroborate the assumption that proteins involved in strict pathogen-host interactions during the infection process are more prone to fix non-synonymous mutations, as previously reported in smaller scale studies (Almeida *et al.* 2012; Borges *et al.* 2012). On the other hand, polymorphism seems to be more pronounced in genes of other functional categories and may be due to discrete genetic drift, as most of the polymorphism is given by dS.

Finally, despite the controversial use of the traditional *ompA*-based typing method, it is worth noting that the main genetic variability within the *C. trachomatis* species is given by the different serovars, where additional strains from the same serovar contribute with few novel polymorphisms (driven either by drift or positive selection) that may impact the individual gene phylogenies (Figure 3).

As concluding remarks, our approach allowed the identification of genes that phylogenetically segregate strains according to specific phenotypes, namely the infection of the ocular tissue, the genitalia, the lymph nodes, as well as their clinical prevalence. It will certainly constitute an important database for prioritizing the targets for functional studies that are mandatory to clarify both their biological role and their involvement in tissue tropism, virulence and ecological success.

## Supplementary Material

Supporting Information
