# Supplementary material for: In Silico Scrutiny of Genes Revealing Phylogenetic Congruence with Clinical Prevalence or Tropism Properties of Chlamydia trachomatis Strains
Source: G3 (Bethesda). 2014 Nov 5;5(1):9–19. doi: 10.1534/g3.114.015354 (PMC4291473; doi:10.1534/g3.114.015354)
Supplement: Supporting Information [file supp_5_1_9__index.html]

In Silico Scrutiny of Genes Revealing Phylogenetic Congruence with Clinical Prevalence or Tropism Properties of Chlamydia trachomatis Strains — Supporting Information 

# *In Silico* Scrutiny of Genes Revealing Phylogenetic Congruence with Clinical Prevalence or Tropism Properties of *Chlamydia trachomatis* Strains

## Supporting Information for Ferreira *et al.*, 2015

**Files in this Data Supplement:**

- Supporting Information - Tables S1-S2, Figure S1, and References (PDF, 357 KB)
- Figure S1 - Nucleotide sequences of crossovers for strains D(s)/2923 and D/SotonD1. (PDF, 299 KB)
- Table S1 - *C. trachomatis* strains used in the present study. (PDF, 167 KB)
- Table S2 - Bioinformatical results of all *C. trachomatis* ORFs with detailed information of putative pseudogenes, strains' segregation, overall mean distances and dN/dS values. (.xlsx, 205 KB)
