# Supplementary material for: In Silico Scrutiny of Genes Revealing Phylogenetic Congruence with Clinical Prevalence or Tropism Properties of Chlamydia trachomatis Strains
Source: G3 (Bethesda). 2014 Nov 5;5(1):9–19. doi: 10.1534/g3.114.015354 (PMC4291473; doi:10.1534/g3.114.015354)
Supplement: Supporting Information [file supp_g3.114.015354_015354SI.pdf]

G3: Genes | Genomes | Genetics

In silico scrutiny of genes revealing phylogenetic congruence with clinical prevalence or tropism properties of *Chlamydia trachomatis* strains

Rita Ferreira *, Minia Antelo *, Alexandra Nunes *,† , Vítor Borges *,†, Vera Damião *, Maria José Borrego *, and João Paulo Gomes *,†,1

* Reference Laboratory of Bacterial Sexually Transmitted Infections and § Bioinformatics Unit, Department of Infectious Diseases, National Institute of Health, Av. Padre Cruz, 1649-016 Lisbon, Portugal

[†] Corresponding author: Department of Infectious Diseases, National Institute of Health, Av. Padre Cruz, 1649-016 Lisbon, Portugal. E-mail address: j.paulo.gomes@insa.min-saude.pt

DOI: 10.1534/g3.114.015354

Table S1 ***C. trachomatis*** **strains used in the present study.**

Strains	Accession nº.	Isolation location	Reference
A/Har13	CP000051	Conjunctiva	Carlson et al. 2005
A/2497	FM872306	Ocular	Harris et al. 2012
A/363	HE601796	Ocular	Harris et al. 2012
A/5291	HE601810	Ocular	Harris et al. 2012
A/7249	HE601797	Ocular	Harris et al. 2012
B/TZ1A828/OT	FM872307	Ocular	Seth-Smith et al. 2009
B/Jali20/OT	FM872308	Ocular	Seth-Smith et al. 2009
C/TW-3	CP006945	Conjunctiva	Borges et al. 2014
D/UW-3/CX	AE001273	Cervix	Stephens et al. 1998
D(s)/2923	ACFJ01000001	Cervix	Jeffrey et al. 2010
D/SotonD1	HE601798	Endocervix	Harris et al. 2012
D/SotonD5	HE601799	Endocervix	Harris et al. 2012
D/SotonD6	HE601800	Endocervix	Harris et al. 2012
E/Bour	HE601870	Ocular	Harris et al. 2012
E/SW2	FN652779	Urethra	Unemo et al. 2010
E/SW3	HE601801	Cervix	Harris et al. 2012
E/SotonE4	HE601802	Endocervix	Harris et al. 2012
E/SotonE8	HE601803	Endocervix	Harris et al. 2012
E/11023	CP001890	Cervix	Jeffrey et al. 2010
E/150	CP001886	Rectum	Jeffrey et al. 2010
F/SW4	HE601804	Cervix	Harris et al. 2012
F/SW5	HE601805	Cervix	Harris et al. 2012
F/SotonF3	HE601806	Endocervix	Harris et al. 2012
F(s)/70	ABYF01000001	Cervix	Jeffrey et al. 2010
G/9301	CP001930	Urethra	Jeffrey et al. 2010
G/9768	CP001887	Rectum	Jeffrey et al. 2010
G/11222	CP001888	Cervix	Jeffrey et al. 2010
G/11074	CP001889	Rectum	Jeffrey et al. 2010
G/SotonG1	HE601807	Endocervix	Harris et al. 2012
J/6276	ABYD01000001	Cervix	Jeffrey et al. 2010
Ia/SotonIa1	HE601808	Endocervix	Harris et al. 2012
Ia/SotonIa3	HE601809	Endocervix	Harris et al. 2012
K/SotonK1	HE601794	Endocervix	Harris et al. 2012
L1/440/LN	HE601950	Lymph node	Harris et al. 2012
L1/1322/p2	HE601951	Genital ulcer	Harris et al. 2012
L1/115	HE601952	LGV patient	Harris et al. 2012
L1/224	HE601953	LGV patient	Harris et al. 2012
L2/434/Bu	AM884176	Lymph node	Thomson et al. 2008
L2/25667R	HE601954	Rectal biopsy	Harris et al. 2012
L2b/UCH-1	AM884177	Rectum	Thomson et al. 2008
L2b/8200/07	HE601795	Rectum	Harris et al. 2012
L2b/UCH-2	HE601956	Rectum	Harris et al. 2012
L2b/Canada1	HE601963	Rectum	Harris et al. 2012
L2b/Canada2	HE601957	Rectum	Harris et al. 2012
L2b/LST	HE601958	Rectum	Harris et al. 2012
L2b/CV204	HE601960	Rectum	Harris et al. 2012
L2b/795	HE601949	Rectum	Harris et al. 2012
L2b/Ams1	HE601959	Penile ulcer	Harris et al. 2012
L2b/Ams2	HE601961	Anus	Harris et al. 2012
L2b/Ams3	HE601962	Anus	Harris et al. 2012
L2b/Ams4	HE601964	Anus	Harris et al. 2012
L2b/Ams5	HE601965	Anus	Harris et al. 2012
L3/404/LN	HE601955	Lymph node	Harris et al. 2012

Table S2 Bioinformatical results of all *C. trachomatis* ORFs with detailed information of putative pseudogenes, strains' segregation, overall mean distances and dN/dS values. The genes' annotation corresponds to the D/UW3-CX strain.

Table S2 is available for download as an Excel file at http://www.g3journal.org/lookup/suppl/doi:10.1534/g3.114.015354/-/DC1

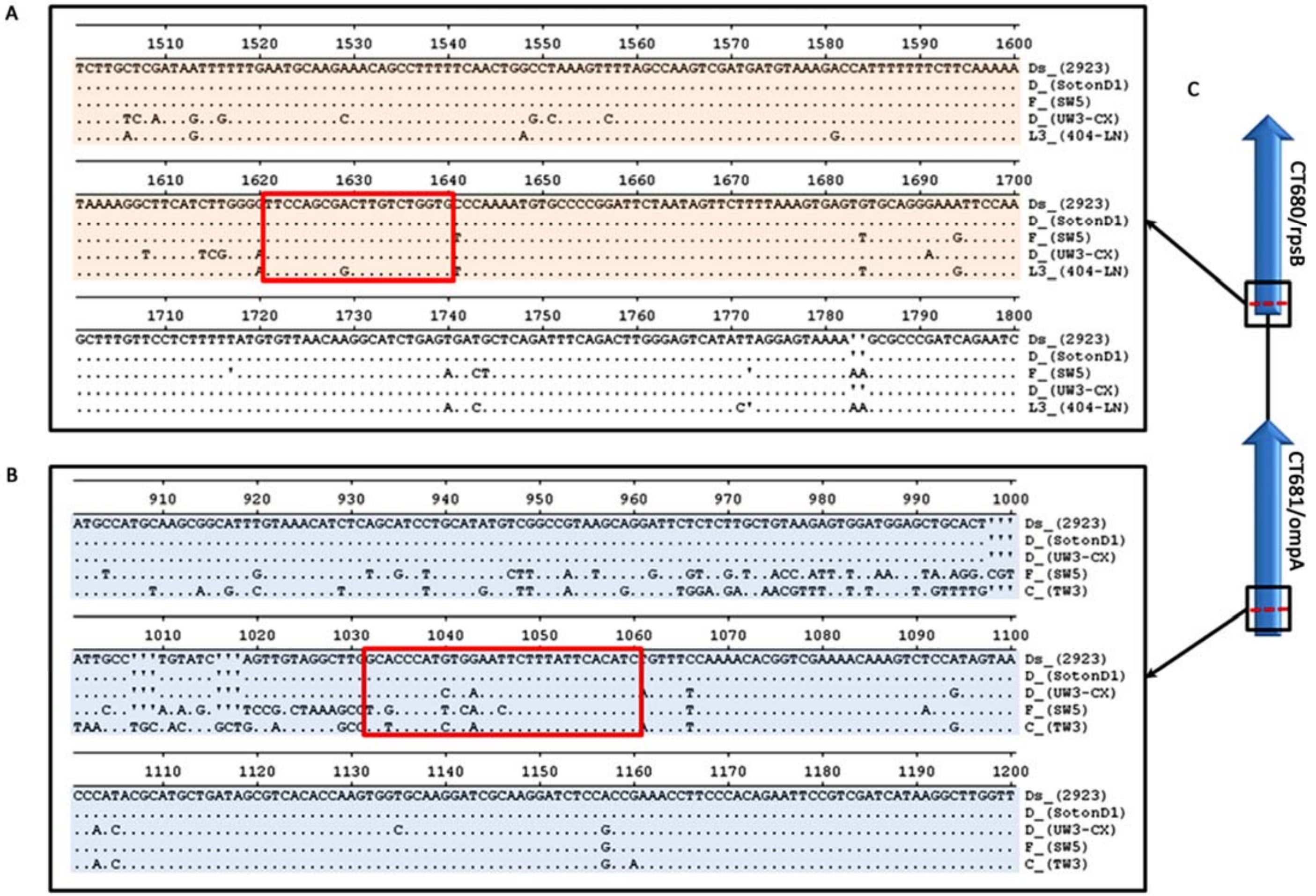

Figure S1 Nucleotide sequences of crossovers for strains D(s)/2923 and D/SotonD1. Crossover regions (red boxes) are delimitated by informative sites from SimPlot/BootScan analysis. Panels A and B represent the partial alignments used for the determination of the crossovers in CT680/*rpsB* and the CT681/*ompA*, respectively. Panel C shows the genetic localization of those partial alignments.

REFERENCES

Borges, V., M. Pinheiro, L. Vieira, D. A. Sampaio, A. Nunes, *et al*., 2014 Complete genome sequence of *Chlamydia trachomatis* ocular serovar C strain TW-3. Genome Announc. 2(1). pii:e01204-13. doi:10.1128/genomeA.01204-13.

Carlson, J.H., S. F. Porcella, G. McClarty, H. D. Caldwell, 2005 Comparative genomic analysis of *Chlamydia trachomatis* oculotropic and genitotropic strains. Infect. Immun. 73:6407-6418.

Harris, S.R., I. N. Clarke, H.M. Seth-Smith, A. W. Solomon, L. T. Cutcliffe, *et al.*, 2012 Whole-genome analysis of diverse Chlamydia trachomatis strains identifies phylogenetic relationships masked by current clinical typing. Nat. Genet. 44:413-419.

Jeffrey, B.M., R. J. Suchland, K. L. Quinn, J. R. Davidson, W. E. Stamm, *et al*., 2010 Genome sequencing of recent clinical Chlamydia trachomatis strains identifies loci associated with tissue tropism and regions of apparent recombination. Infect. Immun. 78:2544-2553.

Seth-Smith, H. M., S. R. Harris, K. Persson, P. Marsh, A. Barron, *et al*., 2009 Co-evolution of genomes and plasmids within *Chlamydia trachomatis* and the emergence in Sweden of a new variant strain. BMC Genomics. 10:239. doi:10.1186/1471-2164-10-239.

Stephens, R.S., S. Kalman, C. Lammel, J. Fan, R. Marathe, *et al*., 1998 Genome sequence of an obligate intracellular pathogen of humans: *Chlamydia trachomatis*. Science. 282:754-759.

Thomson, N. R., M. T. Holden, C. Carder, N. Lennard, S. J. Lockey, *et al*., 2008 *Chlamydia trachomatis*: genome sequence analysis of lymphogranuloma venereum isolates. Genome Res. 18:161-171.

Unemo, M., H. M. Seth-Smith, L. T. Cutcliffe, R. J. Skilton, D. Barlow, *et al*., 2010 The Swedish new variant of Chlamydia trachomatis: genome sequence, morphology, cell tropism and phenotypic characterization. Microbiology 156:139–1404.
