# Supplementary material for: In Silico Scrutiny of Genes Revealing Phylogenetic Congruence with Clinical Prevalence or Tropism Properties of Chlamydia trachomatis Strains
Source: G3 (Bethesda). 2014 Nov 5;5(1):9–19. doi: 10.1534/g3.114.015354 (PMC4291473; doi:10.1534/g3.114.015354)
Supplement: Supporting Information [file supp_g3.114.015354_FigureS1.pdf]

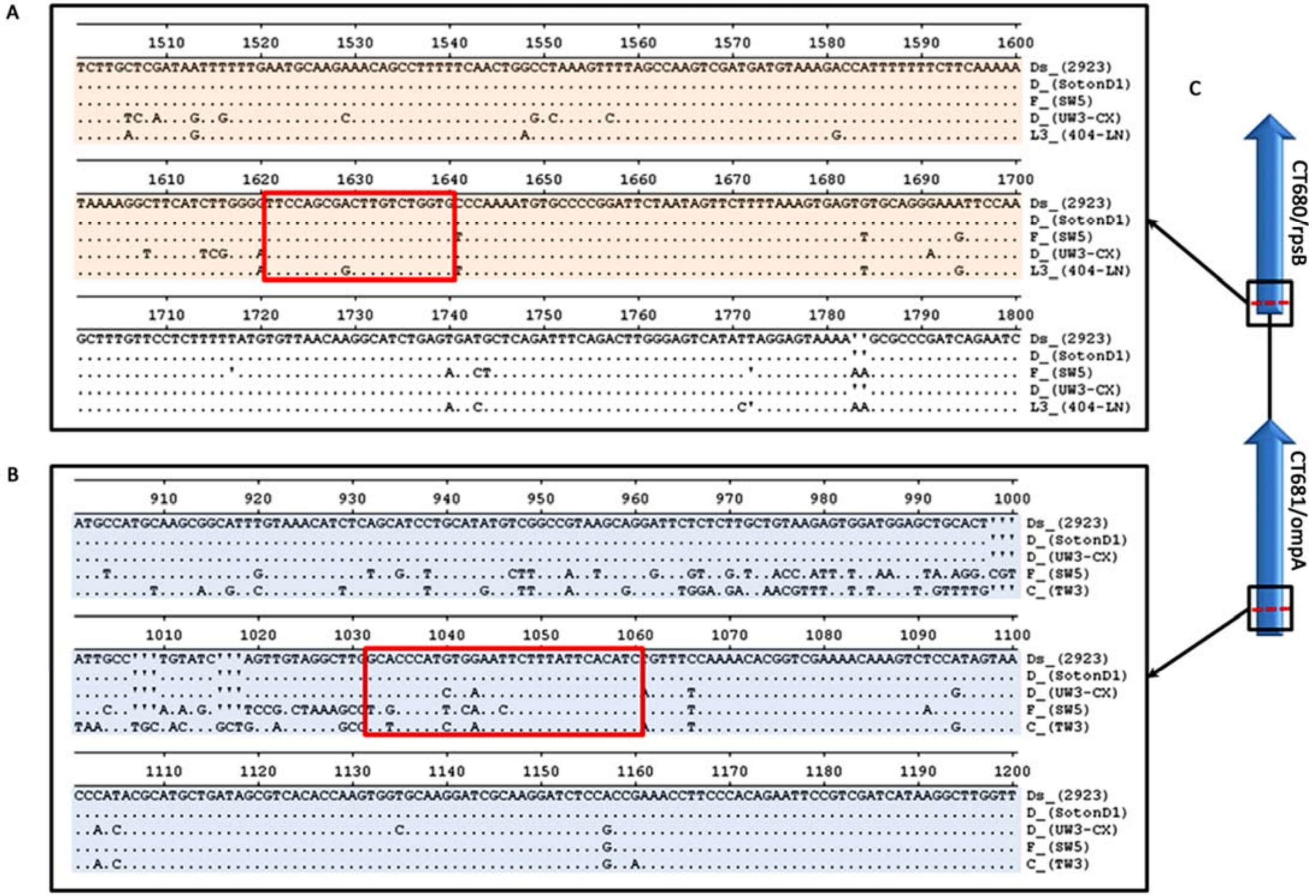


**Figure S1** Nucleotide sequences of crossovers for strains D(s)/2923 and D/SotonD1. Crossover regions (red boxes) are delimitated by informative sites from SimPlot/BootScan analysis. Panels A and B represent the partial alignments used for the determination of the crossovers in CT680/*rpsB* and the CT681/*ompA*, respectively. Panel C shows the genetic localization of those partial alignments.
