# Supplementary material for: In Silico Scrutiny of Genes Revealing Phylogenetic Congruence with Clinical Prevalence or Tropism Properties of Chlamydia trachomatis Strains
Source: G3 (Bethesda). 2014 Nov 5;5(1):9–19. doi: 10.1534/g3.114.015354 (PMC4291473; doi:10.1534/g3.114.015354)
Supplement: Supporting Information [file supp_g3.114.015354_TableS1.pdf]

Table S1 *C. trachomatis* **strains used in the present study.**

Strains	Accession nº.	Isolation location	Reference
A/Har13	CP000051	Conjunctiva	Carlson et al. 2005
A/2497	FM872306	Ocular	Harris et al. 2012
A/363	HE601796	Ocular	Harris et al. 2012
A/5291	HE601810	Ocular	Harris et al. 2012
A/7249	HE601797	Ocular	Harris et al. 2012
B/TZ1A828/OT	FM872307	Ocular	Seth-Smith et al. 2009
B/Jali20/OT	FM872308	Ocular	Seth-Smith et al. 2009
C/TW-3	CP006945	Conjunctiva	Borges et al. 2014
D/UW-3/CX	AE001273	Cervix	Stephens et al. 1998
D(s)/2923	ACFJ01000001	Cervix	Jeffrey et al. 2010
D/SotonD1	HE601798	Endocervix	Harris et al. 2012
D/SotonD5	HE601799	Endocervix	Harris et al. 2012
D/SotonD6	HE601800	Endocervix	Harris et al. 2012
E/Bour	HE601870	Ocular	Harris et al. 2012
E/SW2	FN652779	Urethra	Unemo et al. 2010
E/SW3	HE601801	Cervix	Harris et al. 2012
E/SotonE4	HE601802	Endocervix	Harris et al. 2012
E/SotonE8	HE601803	Endocervix	Harris et al. 2012
E/11023	CP001890	Cervix	Jeffrey et al. 2010
E/150	CP001886	Rectum	Jeffrey et al. 2010
F/SW4	HE601804	Cervix	Harris et al. 2012
F/SW5	HE601805	Cervix	Harris et al. 2012
F/SotonF3	HE601806	Endocervix	Harris et al. 2012
F(s)/70	ABYF01000001	Cervix	Jeffrey et al. 2010
G/9301	CP001930	Urethra	Jeffrey et al. 2010
G/9768	CP001887	Rectum	Jeffrey et al. 2010
G/11222	CP001888	Cervix	Jeffrey et al. 2010
G/11074	CP001889	Rectum	Jeffrey et al. 2010
G/SotonG1	HE601807	Endocervix	Harris et al. 2012
J/6276	ABYD01000001	Cervix	Jeffrey et al. 2010
Ia/SotonIa1	HE601808	Endocervix	Harris et al. 2012
Ia/SotonIa3	HE601809	Endocervix	Harris et al. 2012
K/SotonK1	HE601794	Endocervix	Harris et al. 2012
L1/440/LN	HE601950	Lymph node	Harris et al. 2012
L1/1322/p2	HE601951	Genital ulcer	Harris et al. 2012
L1/115	HE601952	LGV patient	Harris et al. 2012
L1/224	HE601953	LGV patient	Harris et al. 2012
L2/434/Bu	AM884176	Lymph node	Thomson et al. 2008
L2/25667R	HE601954	Rectal biopsy	Harris et al. 2012
L2b/UCH-1	AM884177	Rectum	Thomson et al. 2008
L2b/8200/07	HE601795	Rectum	Harris et al. 2012
L2b/UCH-2	HE601956	Rectum	Harris et al. 2012
L2b/Canada1	HE601963	Rectum	Harris et al. 2012
L2b/Canada2	HE601957	Rectum	Harris et al. 2012
L2b/LST	HE601958	Rectum	Harris et al. 2012
L2b/CV204	HE601960	Rectum	Harris et al. 2012
L2b/795	HE601949	Rectum	Harris et al. 2012
L2b/Ams1	HE601959	Penile ulcer	Harris et al. 2012
L2b/Ams2	HE601961	Anus	Harris et al. 2012
L2b/Ams3	HE601962	Anus	Harris et al. 2012
L2b/Ams4	HE601964	Anus	Harris et al. 2012
L2b/Ams5	HE601965	Anus	Harris et al. 2012
L3/404/LN	HE601955	Lymph node	Harris et al. 2012

Table S2 Bioinformatical results of all *C. trachomatis* ORFs with detailed information of putative pseudogenes, strains' segregation, overall mean distances and dN/dS values. The genes' annotation corresponds to the D/UW3-CX strain.

Table S2 is available for download as an Excel file at http://www.g3journal.org/lookup/suppl/doi:10.1534/g3.114.015354/-/DC1
